# Oral Administration of Silkworm-Produced GAD65 and Insulin Bi-Autoantigens against Type 1 Diabetes

**DOI:** 10.1371/journal.pone.0147260

**Published:** 2016-01-19

**Authors:** Baoping Liu, Yuan Yue, Yun Yang, Yongfeng Jin

**Affiliations:** Institute of Biochemistry, College of Life Sciences, Zhejiang University, 866 Yuhangtang Road, Hangzhou, 310058, China; University of British Columbia, CANADA

## Abstract

Induction of mucosal tolerance by oral administration of protein antigens is a potential therapeutic strategy for preventing and treating type 1 diabetes (T1D); however, the requirement for a large dosage of protein limits clinical applications because of the low efficacy. In this study, we generated a fusion protein CTB-Ins-GAD composed of CTB (cholera toxin B subunit), insulin, and three copies of GAD65 peptide 531–545, which were efficiently produced in silkworm pupae, to evaluate its protective effect against T1D. We demonstrate that oral administration of CTB-Ins-GAD suppressed T1D by up to 78%, which is much more effective than GAD65 single-antigen treatment. Strikingly, CTB-Ins-GAD enhance insulin- and GAD65-specific Th2-like immune responses, which repairs the Th1/Th2 imbalance and increases the number of CD4^+^CD25^+^Foxp3^+^ T cell and suppresses insulin- and GAD65-reactive spleen T lymphocyte proliferation and migration. Our results strongly suggest that the combined dual antigens promote the induction of oral tolerance, thus providing an effective and economic immunotherapy against T1D in combination with a silkworm bioreactor.

## Introduction

Oral tolerance refers to the physiological response of an organism remaining in a state of specific immunological unresponsiveness to orally delivered antigens [1, 2]. Type 1 diabetes (T1D) is a spontaneous organ-specific autoimmune disease [3–5], and a series of autoantigens have been identified in both humans [6, 7] and NOD mice [8], including insulin [6] and glutamic acid decarboxylase (GAD) [8]. Oral administration of these antigens has shown efficacy in preventing T1D in non-obese diabetic (NOD) mice [9–15]. However, the translation of oral tolerance therapies to clinical application remains challenging. Human clinical trials conducted to date using oral immunological tolerance have yielded disappointing results, possibly because of the low efficacy and requirement for large doses of antigen. The overall results of clinical trials showed that oral insulin failed to delay or prevent T1D for the entire study duration [16–19]. However, subgroup analyses revealed that oral insulin delayed diabetes onset for up to 5 years in patients who had high insulin autoantibody levels. Based on these subgroup results, Type 1 Diabetes TrialNet is conducting a new oral insulin prevention trial to verify or refute this observation [20] (www.clinicaltrials.gov/ct2/show/NCT00419562). Another serious limitation in the clinical usage of oral tolerance therapy is the potentially high cost of producing autoantigens, particularly to maintain the beneficial effects for long-term administration [21]. In the case mentioned above, large doses of autoantigens (7.5 mg insulin/day) would limit its clinical application.

In the past 25 years, a series of mucosal adjuvants and cytokines have been used to improve the oral immunological therapeutic efficacy against T1D. In particular, conjugation of GAD or insulin to an adjuvant such as the cholera toxin B subunit (CTB) enhanced the therapeutic efficacy against T1D [10, 13, 22–24]. Furthermore, both interleukin (IL)-4 and human GAD65 plant tissue were required to protect NOD mice from diabetes [21]. In addition, co-administration of human insulin and porcine GAD was more protective than oral GAD alone; however, its suppression efficacy was approximately 25%, much lower than those of the individual antigens [25]. More importantly, it did not induce antigen-specific humoral immune responses or significant transferable suppression, suggesting that other factors may be required to enhance oral tolerance.

Improving the expression level of antigens is another approach for clinical applications. Antigens have been generated in several systems including bacteria [14, 23], yeast [26, 27] and plants [21, 28, 29]. However, the expensive industrial fermentation and elaborate purification methods in bacterial expression systems, as well as the low expression in transgenic plants, limit clinical applications [21, 28, 30]. The silkworm bioreactor offers several advantages for the production of therapeutic proteins [12, 31, 32]. First, silkworms can efficiently express protein, and the expression levels can reach milligram quantities per pupa. Second, silkworms can perform post-translational modifications such as the formation of disulfide bonds, phosphorylation, and glycosylation. Third, silkworm hemolymph is rich in proteinase inhibitors, which may protect recombinant proteins from enzymatic digestion in the gastrointestinal tract. Fourth, the baculovirus is non-infectious in vertebral animals, and the system eliminates concerns regarding pathogens that could potentially be transmitted to humans. Finally, silkworms can be raised on a large scale. We have efficiently expressed proteins and antigens using the silkworm bioreactor [12, 13, 33], some of which have been used for preclinical and clinical trials, including a stage II clinical trial of the first silkworm-based recombinant human granulocyte macrophage colony stimulating factor (rhGM-CSF) oral drug [34]. Therefore, the silkworm system is an ideal expression system for producing mucosal vaccines.

We have previously demonstrated that oral administration of silkworm-produced CTB-insulin was capable of protecting 46% of NOD mice from diabetes [33]. Our present study attempted to improve the prevention efficacy against T1D using a combination of multiple antigens. A fusion protein CTB-Ins-GAD composed of CTB, insulin, and three copies of GAD65 peptide 531–545 was produced in silkworm pupae. We show that oral administration of CTB-Ins-GAD protected NOD mice against diabetes. To our knowledge, this is one of the most effective compounds for the suppression of T1D with high oral tolerance. CTB-Ins-GAD significantly induced both insulin- and GAD65-specific Th2-like immune responses and increased the number of CD4^+^CD25^+^Foxp3^+^ Tregs and suppressed spleen T lymphocyte proliferation and migration. Our results strongly suggest that the dual antigens promote the induction of oral tolerance, thus providing an effective and economic immunotherapy against T1D in combination with a silkworm bioreactor.

## Materials and Methods

### *B*. *mori* pupae, cell line, and mice

Fifth-instar silkworm *B*. *mori* (Jingsong×Haoyue, Showa) were fed fresh mulberry leaves and reared under a photoperiod schedule of 12 h light and 12 h darkness at 25±1°C, after which the silkworm pupae were harvested. BmN cells were from the collections of the College of Life Sciences of Zhejiang University (China) [33] and cultured in TC-100 medium (Gibco-BRL) containing 10% fetal calf serum (Gibco-BRL) and 50 μg/ml gentamycin at 27°C. Female NOD mice and NOD severe combined immunodeficient mice (NOD/SCID) were purchased from the Shanghai Laboratory Animal Center, Chinese Academy of Sciences (SLAC, CAS, China), and housed at the animal central of the medical department of Zhejiang University where they were screened for bacterial and viral pathogens. The animals' care was in accordance with institutional guidelines of Laboratory Animals of the National Institutes of Health. The protocol was approved by the Committee on the Ethics of Animal Experiments of the medical department of Zhejiang University.

### Acquisition of recombinant baculovirus in BmN cells

Seven primers were designed to construct the CTB-Ins-GAD and CTB-GAD-Ins fusion genes (S1 Table), after which the fusion genes were subcloned into the donor pFastBac1 plasmid to construct the recombinant bacmids CTB-Ins-GAD and CTB-GAD-Ins. A subconfluent monolayer of BmN cells was transfected with recombinant bacmid CTB-Ins-GAD and CTB-GAD-Ins using Lipofectamine 2000 (Invitrogen, USA). The recombinant virus was generated in transfected BmN cells after 3–5 days and was then used to infect BmN cells and silkworm pupae for fusion protein expression.

### Expression and collection of the fusion proteins

BmN cells (2×10^6^) were infected with BmNPV CTB-GAD-Ins and BmNPV CTB-Ins-GAD at a multiplicity of infection (MOI) of 10 and collected 2–7 days after infection. Harvested BmN cells were suspended in 0.5 ml phosphate-buffered saline (PBS) and lysed by gentle sonication several times on ice. The cells were centrifuged, and the supernatant was removed and stored at -20°C. The silkworm pupae were needle inoculated with the viral solutions (1×10^7^ pfu/ml) into their body cavities. The silkworm pupae were collected at 2–7 days after inoculation and stored at -20°C.

### Western blot analysis and ELISA

The detection and quantification of CTB-GAD-Ins and CTB-Ins-GAD fusion proteins were performed as described previously [13]. The cell lysate or hemolymph samples were diluted and separated by 12% SDS-PAGE. Samples were either boiled or loaded directly on the gel. Rabbit anti-cholera toxin (Sigma, USA) and rabbit anti-insulin primary antibodies (Epitomics, USA) were used for the immunoreactions.

A semiquantitative ELISA was used to investigate the expression level of the fusion protein. A 96-well microtiter plate was loaded with dilutions of the cell-lysed supernatant (1:20–1:100) or hemolymph (1:500–1:2000) in bicarbonate buffer, pH 9.6 (15 mM Na_2_CO_3_, 35 mM NaHCO_3_) at 4°C overnight. Serial dilutions of bacterial CTB (Sigma, USA) were used to generate the standard curve to calculate the results. The plate was blocked with 1% BSA at 37°C for 1 h and then washed with PBS containing 0.05% Tween-20 (PBST). A 1:8000 dilution of anti-cholera toxin antibody in 1% BSA was added at 37°C for 2 h. It was then washed with PBST and incubated with a 1:10000 dilution of anti-rabbit IgG conjugated with horseradish peroxidase at 37°C for 40 min. Finally, the chromogenic substrate O-phenylenediamine was added at 37°C for 30 min to develop color, and 2 M H_2_SO_4_ (50 μl/well) was added to stop the reaction. The absorbance at 492 nm was measured in a Labsystems Multiscan MS ELISA plate reader (Labsystems, Finland).

### GM1 ganglioside-binding assay

A GM1-ELISA was performed as described previously [13] to detect the affinity of silkworm-derived fusion proteins for GM1 ganglioside. The microtiter plates were coated with monosialoganglioside-GM1 (Sigma, USA) by incubating the plates with 50 μl/well GM1 in methanol at 4°C overnight. The wells were then blocked with BSA solution, and the hemolymph dilutions were added. The same dilutions of normal hemolymph and serial dilutions of bacterial CTB were used as a negative control and to generate the standard curve, respectively. The remainder of the procedure was identical to the semiquantitative ELISA assay described above.

### Induction of oral tolerance

Five-week-old female NOD mice were divided into four groups and fed wild-type (WT) silkworm pupae hemolymph, silkworm pupae hemolymph synthesized CTB-GAD protein, CTB-Ins-GAD protein, or CTB-GAD-Ins protein. Beginning at 5 weeks of age, the mice were delivered an equal amount of hemolymph by intragastric administration (~100–300 μl) every other day until 10 weeks of age. Each feeding of hemolymph delivered approximately 10 μg of the corresponding fusion protein. The animals were sacrificed at 10 weeks of age for antibody titer assays and pancreatic islet histopathological analysis.

### Pancreatic islet histopathological analysis

To evaluate insulitis in experimental NOD mice, the extent of lymphocyte infiltration in the islets was measured. At 10 weeks of age, six mice in each group were sacrificed, and the pancreas was removed for insulitis investigation. The degree of insulitis was evaluated by two independent observers using a standardized scoring system with a semiquantitative scale ranging from 0 to 4: 0, normal islets with no sign of T-cell infiltration; 1, focal peri-islet T-cell infiltration; 2, more extensive peri-islet infiltration but with lymphocytes less than one-third of the islet area; 3, intraislet T-cell infiltration in one-third to one-half of the islet area; and 4, extensive intraislet inflammation involving more than half of the islet area. At least 25 islets were scored for each animal.

### Assessment of diabetes

Incidences of diabetes were compared among mice fed the CTB-GAD, CTB-Ins-GAD, or CTB-GAD-Ins fusion proteins or WT. The feeding schedule was the same as mentioned above and continued for 30 weeks. Starting at 10 weeks of age, the development of diabetes was monitored and confirmed by measuring blood glucose using the ACCU-CHEK III system (Roche Diagnostics Ltd., Shanghai, China). A mouse with a blood glucose level above 8.0% (200 mg/dL) for 2 consecutive weeks was considered diabetic. For remission studies, female NOD mice at age 16–24 weeks were treated with oral administration of CTB-GAD, CTB-Ins-GAD, CTB-GAD-Ins or WT silkworm pupae hemolymph once they were confirmed to have blood glucose levels between 200 and 250 mg/dl. Ten micrograms of the corresponding fusion protein was administered every day on days 1–7 and then twice a week for 50 days. Blood glucose levels were monitored twice a week for 50 days and mice having blood glucose levels >600 mg/dl or >400 mg/dl for four consecutive measurements were considered to have reached the study endpoint.

### Quantification of serum antibody subtypes

Five-week-old female NOD mice were fed CTB-Ins-GAD, CTB-GAD-Ins, CTB-GAD, or WT alone three to four times per week until 10 weeks of age. Treated NOD mouse serum was quantified for anti-CTB, anti-insulin or anti-GAD65 antibodies and antibody subtypes using ELISA. Human insulin (Novo Nordisk), GAD65, or bacterial CTB was used as the well-coating antigen, and after blocking, serial dilutions of pooled sera was added to the coated microtiter plate wells. Horseradish peroxidase-conjugated anti-mouse IgG1, IgG2a, IgA, or IgE antibody (SABC, China) was used as the secondary antibody. The absorption value was measured as described above. The titer was defined as the reciprocal of the highest dilution of the sample giving an absorption signal above background, and it was individually determined for each sample.

### ELISPOT assays

ELISPOT assays were performed using antibody pairs for mouse IFN-γ and IL-4 (BioLegend), as indicated by the manufacturer. Splenic cell suspensions prepared from the 10-week-old fusion protein-fed NOD mice used for antibody quantification were added to individual wells (1×10^6^ cells/well) alone or with insulin and GAD65 as antigens (20 μg/ml) and incubated for 72 h. Biotinylated anti-mouse IFN-γ and IL-4 detection antibody (1 μg/ml, BD Pharmingen Inc., San Diego, CA, USA) were added to the plates and incubated overnight. The plate-bound secondary antibody was visualized using horseradish peroxidase and 3-amino-9-ethylcarbazole (AEC) substrate (both from Sigma-Aldrich, St. Louis, MO, USA). The brown colored immunospots corresponding to the cells secreting IFN-γ and IL-4 were counted manually under a dissection microscope. Results are expressed as numbers of spots per 10^6^ splenic cells after subtraction of the background spots appearing in non-antigen-stimulated cultures.

### Cytometric analysis of Treg cell flow

Lymphocytes in the pancreas and pancreatic lymph nodes (PLN) of 10-week-old treated NOD mice were obtained using the help of mouse lymphocyte separation medium (Dakewe Biotech Company, Limited, China). The assay was performed using a mouse regulatory T-cell staining kit containing APC-antiCD3, FITC-antiCD4, PE-antiCD25 and PE-cy5-antiFoxp3 antibodies, according to the manufacturer’s protocol. Proportions of Treg cells in the lymph tissues were analyzed using flow cytometric analysis of lymphocyte phenotypic markers using a FACScan flow cytometer (BeckmanCoulter, USA).

### Spleen lymphocyte proliferation analysis

NOD mice were sacrificed and subjected to splenectomy after 5 weeks of treatment. Spleen lymphocytes were recovered as a monodisperse suspension in RPMI 1640 medium. Erythrocytes were removed from splenic suspensions by lysis in NH_4_Cl for 2 min at 37°C. Cells were cultured in 96-well plates at 2×10^6^/well with 20 μg/ml insulin, GAD65 or 5μg/ml PHA for 2 d at 37°C in 5% CO_2_ air, then they were treated 10 μM 5-bromo-2-deoxyuridine for 1 d at 37°C in 5% CO_2_ air. They were then fixed and treated with Triton X-100 and H_2_O_2_, denatured with HCl, and neutralized with boric acid at room temperature. Following blocking, the cells were treated with a mouse anti-BrdU antibody (Boster, China) and then incubated with a biotin-labeled anti-mouse IgG antibody. Next, ABC compound was added to the cells. DAB was used to develop the corresponding brown color with hematoxylin used as a counterstain. Samples were analyzed using a microscope at 400× magnification. The total cells and the cells with brown color (proliferation cells) were numbered. Ten different regions per samples were analyzed under the microscope. The result was expressed as proliferation cells ratio (proliferation cells / total cells).

### Spleen lymphocyte migration assay

Spleen lymphocytes (1×10^5^) were isolated from NOD mice and cultured in the upper chamber of 24-well Transwell plates with RPMI 1640 serum-free medium. Insulin (20 μg/ml), GAD65 (20 μg/ml) and IL-2 (150 IU/ml) was added to the normal culture medium and placed in the bottom chamber, and the cells were cultured for ~14 h at 37°C with 5% CO_2_. The insulin-reactive and GAD65-reactive T cell will migrate towards insulin and GAD65. Next, the cells were fixed and stained with crystal violet solution after the cells on the chamber were clear. Samples were analyzed using a microscope at 400× magnification.

### Adoptive transfer of diabetes

Splenocytes from 10-week-old NOD mice fed CTB-Ins-GAD, CTB-GAD-Ins, CTB-GAD fusion proteins or WT were assessed for their capacity to suppress the diabetogenic activity of splenocytes obtained from an acutely diabetic donor. Briefly, splenocytes (1×10^7^) from the four treated groups were mixed with splenocytes (1×10^7^) from newly diabetic NOD mice and administered by intravenous injection into the tail veins of 6-8-week-old NOD/SCID mice. Mice receiving only 1×10^7^ diabetic splenocytes were used as a control. Recipient mice were monitored for the development of diabetes for 18 weeks.

### Statistical analysis

Survival analyses with Kaplan-Meier estimates were used to evaluate the difference in diabetes incidence among the NOD mice groups, with the differences being determined by log rank analysis. Adoptive transfer was analyzed using Wilcoxon’s rank-sum test and Fischer’s exact test analysis. Otherwise, results were analyzed using t-tests or ANOVA for multiple comparisons. A P value less than 0.05 was considered to indicate statistical significance in all cases.

## Results

### Recombinant CTB-Ins-GAD expressed in silkworm

**Fig 1:**
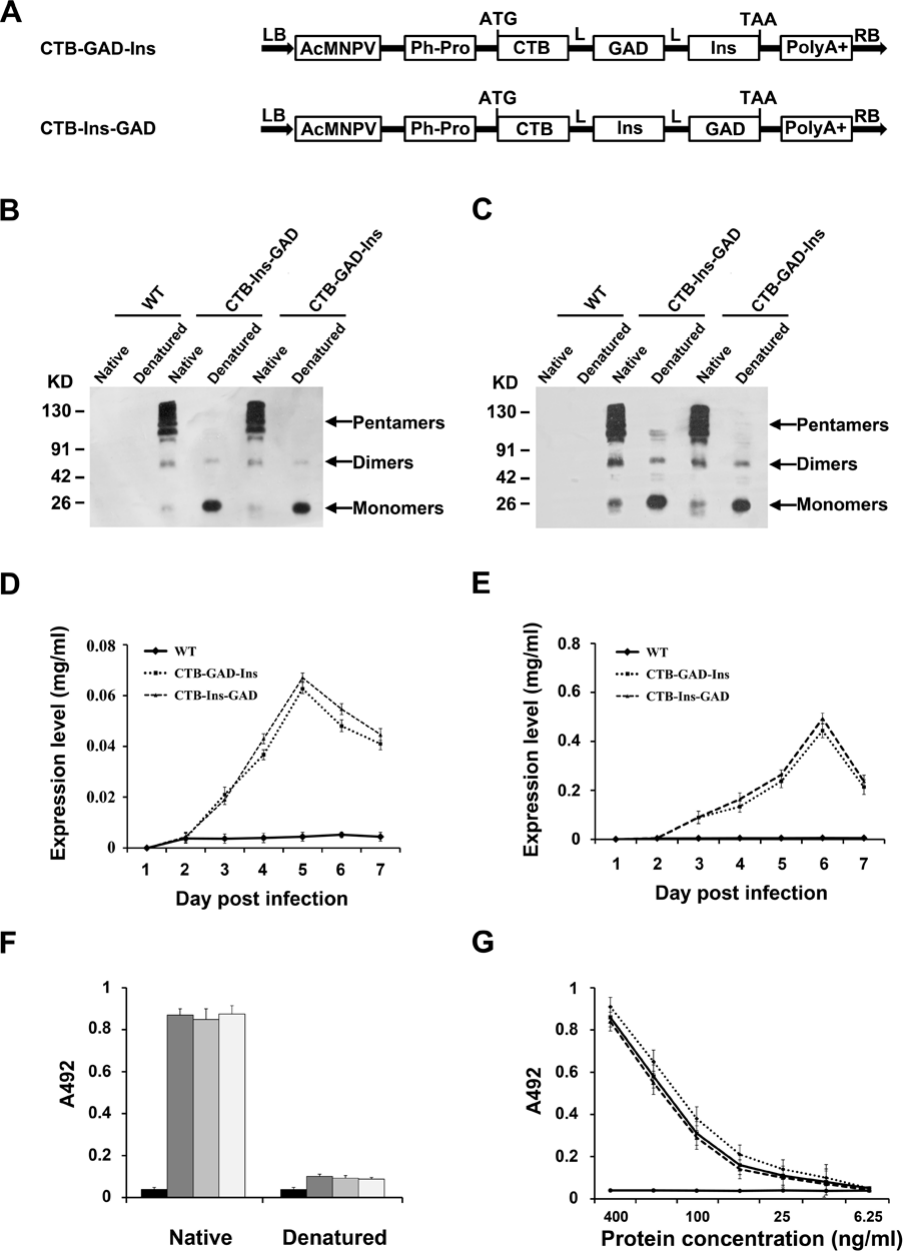
Identification of recombinant CTB-Ins-GAD and CTB-GAD-Ins expressed in silkworm. (A) Schematic structure of the CTB-Ins-GAD fusion gene. AcMNPVPh-Pro: Autographa californica multiple nuclear polyhedrosis virus polyhedrin promoter, CTB: cholera toxin B subunit, L: linker peptide (GPGP), Ins: human insulin, GAD: triple copies of the glutamic acid decarboxylase 65 epitope (GAD65_531-545_), and LB and RB: left and right border, respectively, of the donor WT pFastBac1. The hemolymph from silkworm pupae was analyzed for the expression of the CTB-Ins-GAD fusion protein using anti-CTB (B) or anti-insulin (C) primary antibodies. The boiled and unboiled samples were diluted 50- and 10-fold, respectively. Quantitative analysis of protein production in BmN cells (D) and silkworm pupae (E). Data are presented as the mean concentrations ± standard deviations (SD) on each day. (F) Reactivity of fusion protein with the GM1 ganglioside and a native bacterial CTB control. (G) Pentamer and monomers analyzed. Approximately equal amounts of each sample were used to measure A_492_ signal levels. Data represent the mean A_492_ value ± SD of each sample.

To determine whether the combined oral administration of insulin and GAD65 promoted protection against T1D, we constructed the CTB-Ins-GAD fusion gene (Fig 1A). To decrease the size of the CTB-fused protein to improve its expression level and oral immunology tolerance, we selected three copies of the GAD65 peptide 531–545 instead of the entire GAD65 protein. A flexible hinge tetrapeptide (GPGP) was introduced among the three peptides to maintain the natural conformation of insulin, GAD65, and CTB. The CTB-Ins-GAD and CTB-GAD-Ins fusion genes were inserted into the baculovirus genome immediately downstream of the polyhedron promoter (Fig 1A). We constructed recombinant baculovirus BmNPV-CTB-Ins-GAD, CTB-GAD-Ins and expressed it in silkworm cells and pupa. Western blot analysis using anti-CTB (Fig 1B) or anti-Ins (Fig 1C) antibodies indicated that the CTB-Ins-GAD and CTB-GAD-Ins fusion proteins are ∼130 kDa, while a specific band with a molecular weight of ∼26 kDa was observed (protein) after boiling treatment. This indicated that the recombinant protein existed as a pentamer. Moreover, CTB-Ins-GAD was expressed in BmN cells at 0.037 mg/1×10^6^ cells on day 5 post-infection (Fig 1D). In infected silkworm pupae, the recombinant proteins were efficiently produced at a maximum level of 0.49 mg/ml (Fig 1E).

To investigate the specific affinity of the pentameric protein, we used a GM1-ELISA method with GM1-ganglioside as the capture molecule and bacterial pentameric CTB to produce a standard curve. A concentration-specific absorption signal was observed in CTB-Ins-GAD, similar to bacterial pentameric CTB, which indicates that the recombinant protein exhibited a high affinity for GM1 ganglioside necessary to enhance oral immunological tolerance (Fig 1F and 1G). In addition, similar biochemical and antigenic results have been obtained for CTB-GAD-Ins, in which the order of insulin and GAD65 administration was reversed (Fig 1A). This suggests that the order of administration of insulin and GAD65 has little effect on the biochemical and antigenic properties.

### Oral administration of CTB-Ins-GAD and CTB-GAD-Ins suppressed insulitis and diabetes

**Fig 2:**
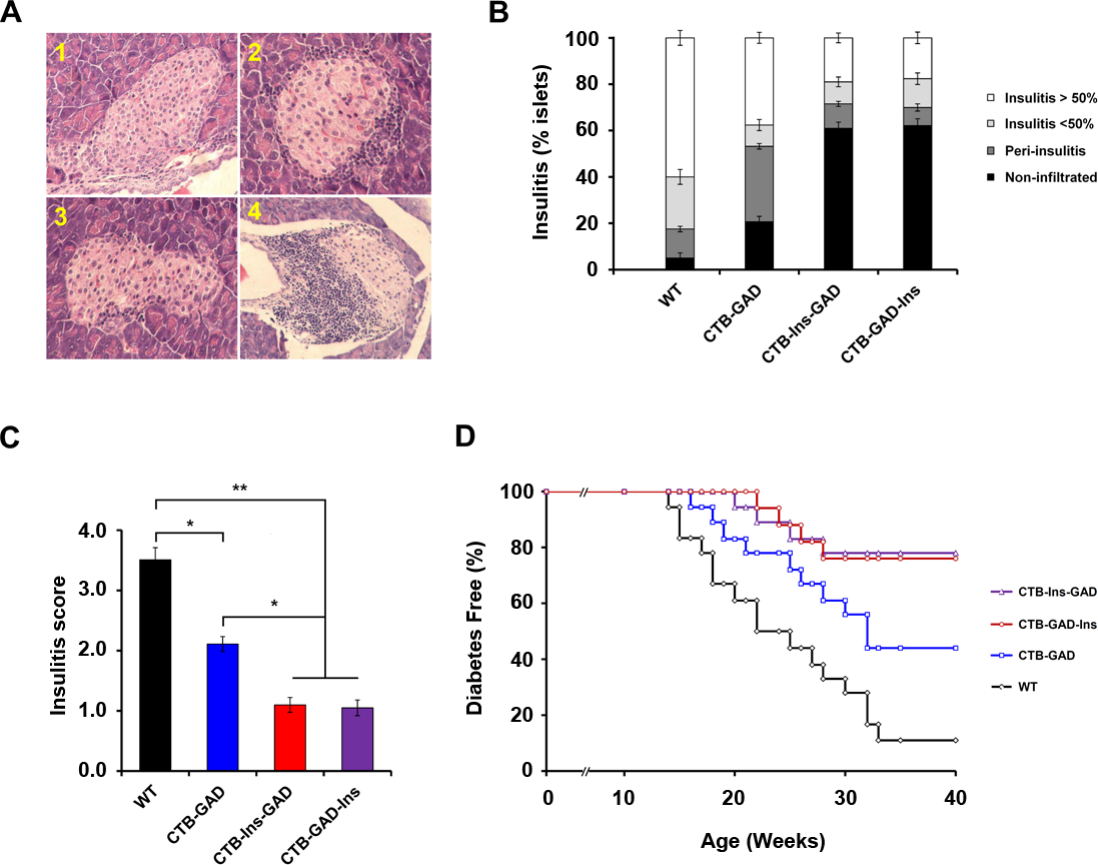
Suppression of insulitis and diabetes. (A) Representative histopathological pancreatic islets from normal mice or experimental NOD mice, more than 25 pancreatic islets per mouse were scored, 1–4: normal mice (n = 6), CTB-GAD (n = 6), CTB-Ins-GAD (n = 6) and WT treated mice (n = 6), respectively. (B and C) Semiquantitative analysis of pancreatic islet insulitis scores. Six mice per group were individually tested in two separate experiments. * p<0.05; ** P<0.01. Data represent the mean values ± SD of each sample. (D) Suppression of diabetes. Five-week-old female NOD mice were fed CTB-Ins-GAD (n = 18), CTB-GAD-Ins (n = 17), CTB-GAD (n = 18), or WT (n = 18) alone three to four times per week until 35 weeks of age. Diabetes was confirmed by hyperglycemia (>200 mg/dL glucose) for 2 consecutive weeks. Data are combined from two independent experiments.

To determine the extent to which oral administration of CTB-Ins-GAD and CTB-GAD-Ins enhances the suppression of insulitis, we compared insulitis reduction among animals fed silkworm-expressed CTB-Ins-GAD, CTB-GAD-Ins, CTB-GAD, or the control. After 5 weeks of supplementation, their pancreatic tissues were harvested, and the islets of each animal were scored (Fig 2A). As a result, less than 20% of the examined islets showed severe insulitis in mice fed CTB-Ins-GAD, while more than 40% showed severe insulitis in mice fed CTB-GAD (Fig 2B). Moreover, the mice fed CTB-Ins-GAD showed a more pronounced decrease in insulitis compared with the CTB-GAD-fed mice (1.05±0.12 vs. 2.11±0.13, p<0.05) (Fig 2C). Similar results were obtained with the CTB-GAD-Ins treatment. Overall, this result shows that CTB-Ins-GAD has an enhanced effect on the suppression of insulitis compared with CTB-GAD.

We next assessed the long-term effects of oral administration of CTB-Ins-GAD and CTB-GAD-Ins on the prevention of diabetes. Pre-diabetic female NOD mice were orally administered CTB-Ins-GAD and CTB-GAD-Ins from 5 to 35 weeks of age. Consequently, the incidence of diabetes decreased significantly in the group fed CTB-Ins-GAD, CTB-GAD-Ins or CTB-GAD compared with the control group. For the whole period from 26 weeks to 35 weeks of age the incidence of diabetes was significantly lower in the CTB-Ins-GAD group than that in the CTB-GAD group. At 35 weeks of age, only 4 out of 18 (22%) mice fed CTB-Ins-GAD developed diabetes, as compared with 10 out of 18 (55%) mice fed CTB-GAD developed diabetes (p<0.05) (Fig 2D). Together with our previous study, which showed only a 46% [33] and 30% [13] suppression of diabetes in mice fed CTB-insulin at 35 weeks of age, as compared with 62% suppression of diabetes in mice fed CTB-Ins-GAD (62% vs 46%, p<0.05; 62% vs 30%, p<0.05). The similar results were obtained with the CTB-GAD-Ins treatment. Thus, CTB-Ins-GAD and CTB-GAD-Ins are much more effective for the suppression of diabetes than insulin or GAD65 single-antigen treatment.

### Oral administration of CTB-Ins-GAD and CTB-GAD-Ins reverse diabetes

**Fig 3:**
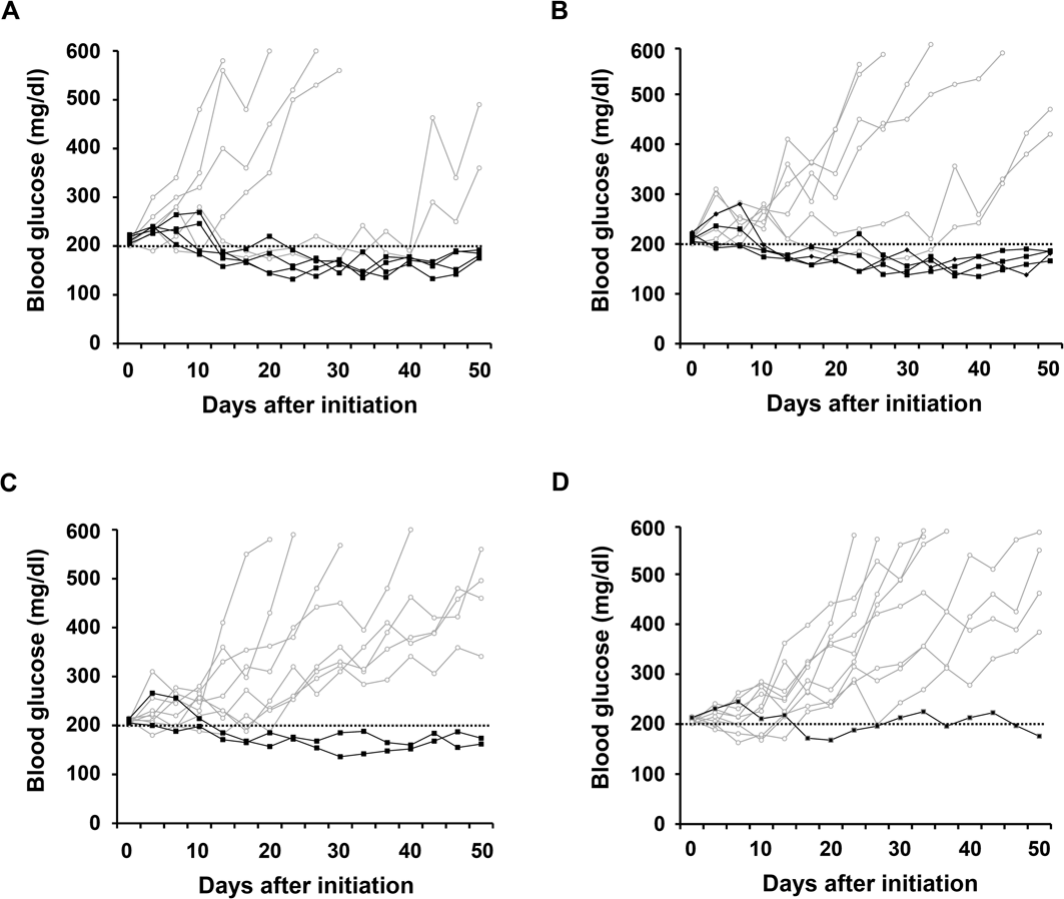
Diabetes reverse assay. Blood glucose levels in the NOD mice treated with (A) CTB-Ins-GAD (n = 10), (B) CTB-GAD-Ins (n = 10), (C) CTB-GAD (n = 10), or (D) WT (n = 10) alone were monitored twice a week until 50 days starting on the onset of diabetes with glucose levels between 200 mg/dl and 249 mg/dl. The mice that developed endpoint are shown as open circles (○) and others are shown as filled squares (■).

We also evaluated the efficacy of CTB-Ins-GAD fusion protein to reverse diabetes after the onset of hyperglycemia. Our results showed that immunization with CTB-Ins-GAD fusion protein resulted in remission from hyperglycemia in 60% of mice, which is more often than the CTB-GAD fusion protein or WT-treated mice within 40 days of initial hyperglycemia (p<0.05) (Fig 3). The protective effect waned after 40 days of initial hyperglycemia. The similar results were obtained with the CTB-GAD-Ins treatment. In contrast, no significant remission of hyperglycemia was observed in the CTB-GAD fusion protein immunized mice compared with WT immunized mice. Thus, this result indicated that CTB-Ins-GAD could temporarily reverse diabetes.

### Oral administration of CTB-Ins-GAD and CTB-GAD-Ins induced both GAD65- and insulin-specific Th2-like immune responses

**Fig 4:**
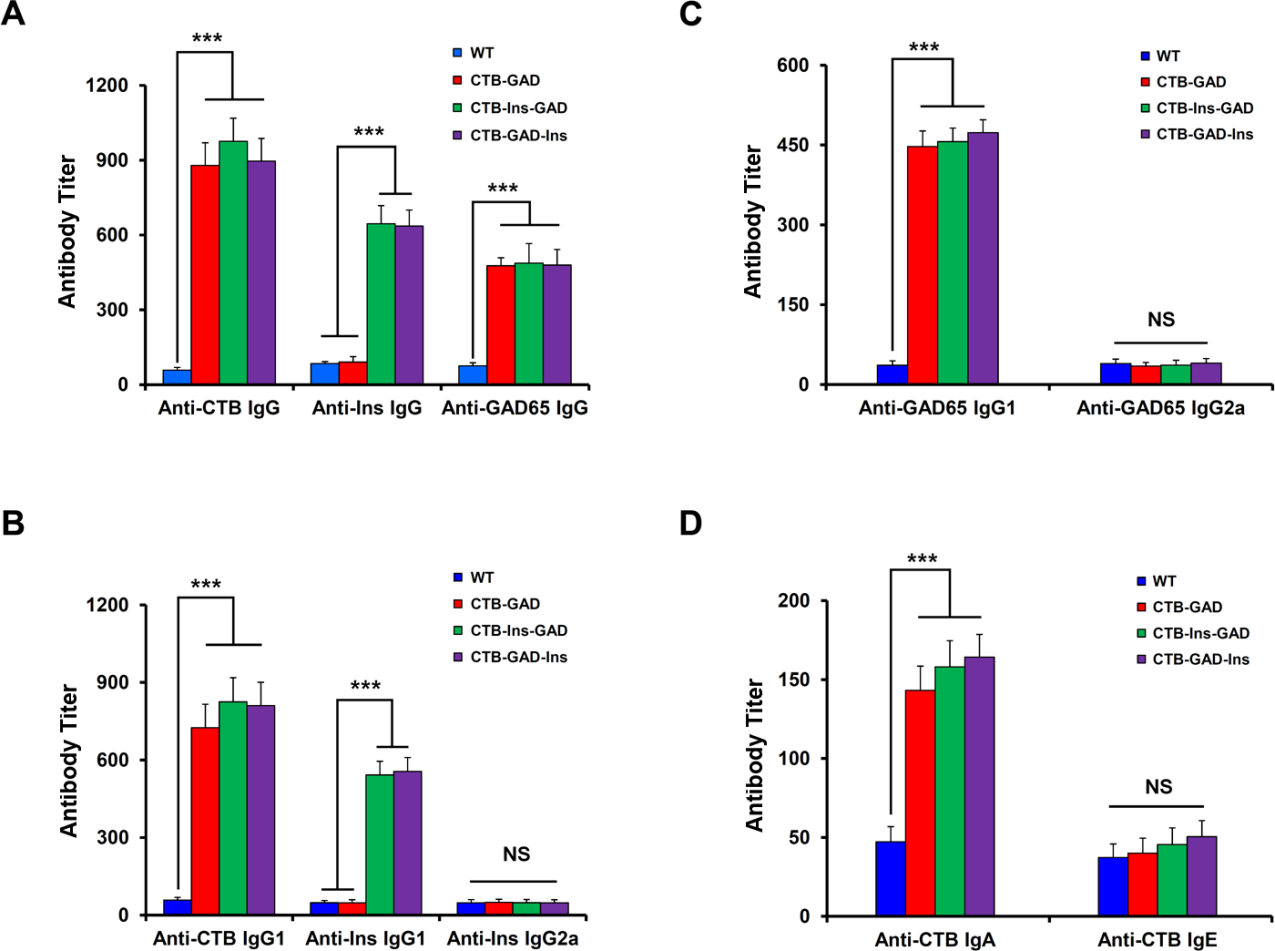
Serum antibodies in NOD mice with different treatments. Five-week-old female NOD mice were fed CTB-Ins-GAD (n = 6), CTB-GAD-Ins (n = 6), CTB-GAD (n = 6), or WT (n = 6) alone three to four times per week until 10 weeks of age. The serum antibodies were detected. (A) Anti-CTB, anti-insulin, anti-GAD65 IgG serum antibody titers; (B) anti-CTB IgG1, anti-insulin IgG1, and anti-insulin IgG2a; (C) anti-GAD65 IgG1, anti-GAD65 lgG2a; (D) anti-CTB IgA and IgE serum titers in mice fed CTB-Ins-GAD, CTB-GAD-Ins, CTB-GAD, or WT. Results are presented as the mean titer values ± SD. Six mice per group were individually tested in two separate experiments. *** P<0.001; NS: no significant difference.

To examine the mechanism underlying the combined effect on the suppression of insulitis, we first compared serum-specific antibodies and their subtype levels across various groups. Our results show that mice immunized with CTB-Ins-GAD produced high titers of anti-Ins and anti-GAD65 antibodies, while those immunized with CTB-GAD produced only the anti-GAD65 antibody (Fig 4A). As expected, similar serum anti-GAD65 IgG1 levels were observed in CTB-Ins-GAD- and CTB-GAD-treated mice (Fig 4C). However, serum anti-Ins IgG1 levels were significantly higher in animals fed CTB-Ins-GAD than those fed CTB-GAD (Fig 4B). By contrast, no significant difference in serum anti-GAD65 or anti-Ins IgG2a was observed between the groups (Fig 4B and 4C). In addition, enhanced serum anti-CTB IgA was observed in the CTB-Ins-GAD and CTB-GAD groups (Fig 4D). The similar results were obtained with the CTB-GAD-Ins treatment. Collectively, these results indicated that mice immunized with CTB-Ins-GAD and CTB-GAD-Ins induced both GAD65- and insulin-specific Th2-like (IgG1), but not Th1-like (IgG2a), humoral immune responses.

### Oral administration of CTB-Ins-GAD and CTB-GAD-Ins induced an increased Th2 shift from the Th1 profile

**Fig 5:**
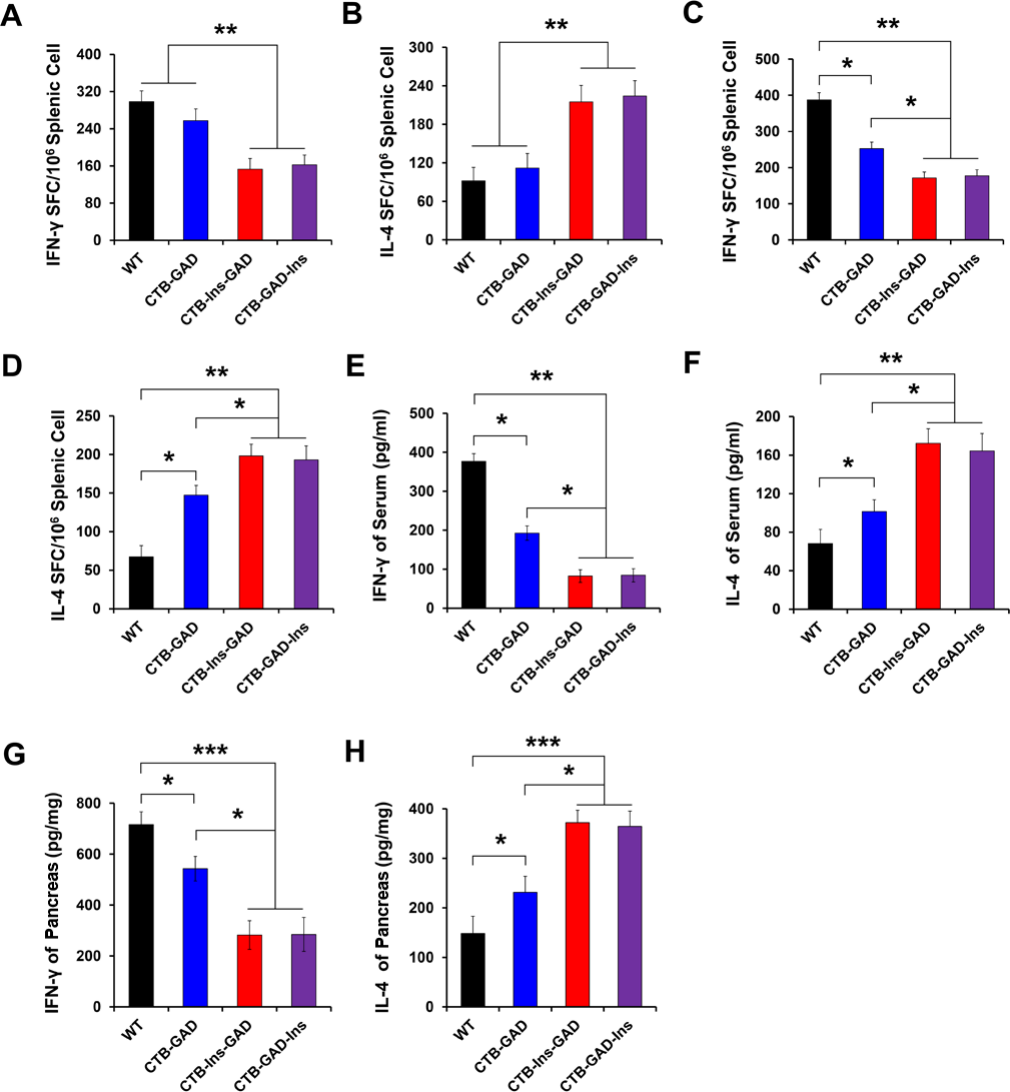
ELISPOT analysis of IL-4/IFN-γ-producing T cells and cytokine assay. Splenocytes from the same 10-week-old NOD mice used to examine the development of insulitis were pooled into a given treatment group. In the ELISPOT assay, splenocytes (10^6^/well) from CTB-Ins-GAD-, CTB-GAD-Ins-, CTB-GAD-, or WT-immunized mice were stimulated with insulin (A and B) or GAD65 peptide (C and D). T cells producing IL-4 (B and D) or IFN-γ (A and C) were detected. The number of spots was quantified in triplicate wells for each group. IFN-γ or IL-4 concentrations in serum (E and F) and pancreas (G and H) was measured via ELISA. Six mice per group were individually tested in two separate experiments. All data are expressed as the means ± SD. * p<0.05; ** P<0.01; *** P<0.001.

To determine how the enhanced suppressive effect on diabetes was mediated by Th2 cells in CTB-Ins-GAD-treated mice, we investigated the frequency of IFN-γ-secreting T cells (Th1) and IL-4-secreting T cells (Th2) using ELISPOT assays. We observed a significant increase in Th2 cells (albeit with a decrease in Th1 cells) in response to stimulation with insulin in the CTB-Ins-GAD group, but not in the CTB-GAD group (Fig 5A and 5B). Importantly, when stimulated with GAD65, we found that the number of Th2 cells was significantly enhanced, while the number of Th1 cells was significantly reduced in the CTB-Ins-GAD group compared with the CTB-GAD group (Fig 5C and 5D). The similar results were obtained with the CTB-GAD-Ins treatment. These results indicate that oral administration of CTB-Ins-GAD induced Th2 cells and suppressed Th1 cells.

We further measured the production of IFN-γ and IL-4 cytokines associated with Th1 and Th2 responses, respectively, in the serum and pancreas of NOD mice. Our results showed that oral administration of CTB-Ins-GAD led to a stronger induction of serum and pancreas IL-4 than that of CTB-GAD (Fig 5F and 5H). Conversely, CTB-Ins-GAD treatment significantly decreased IFN-γ levels compared with CTB-GAD (Fig 5E and 5G). The same result was observed in the CTB-GAD-Ins group. The enhanced IL-4 and reduced IFN-γ induced by CTB-Ins-GAD further supported the ELISPOT results. Overall, these results indicated that CTB-Ins-GAD can induce a shift not only in Th1/Th2 cells, but also in Th2 cytokine production in NOD mice.

### Oral administration of CTB-Ins-GAD and CTB-GAD-Ins enhanced regulatory T cell differentiation

**Fig 6:**
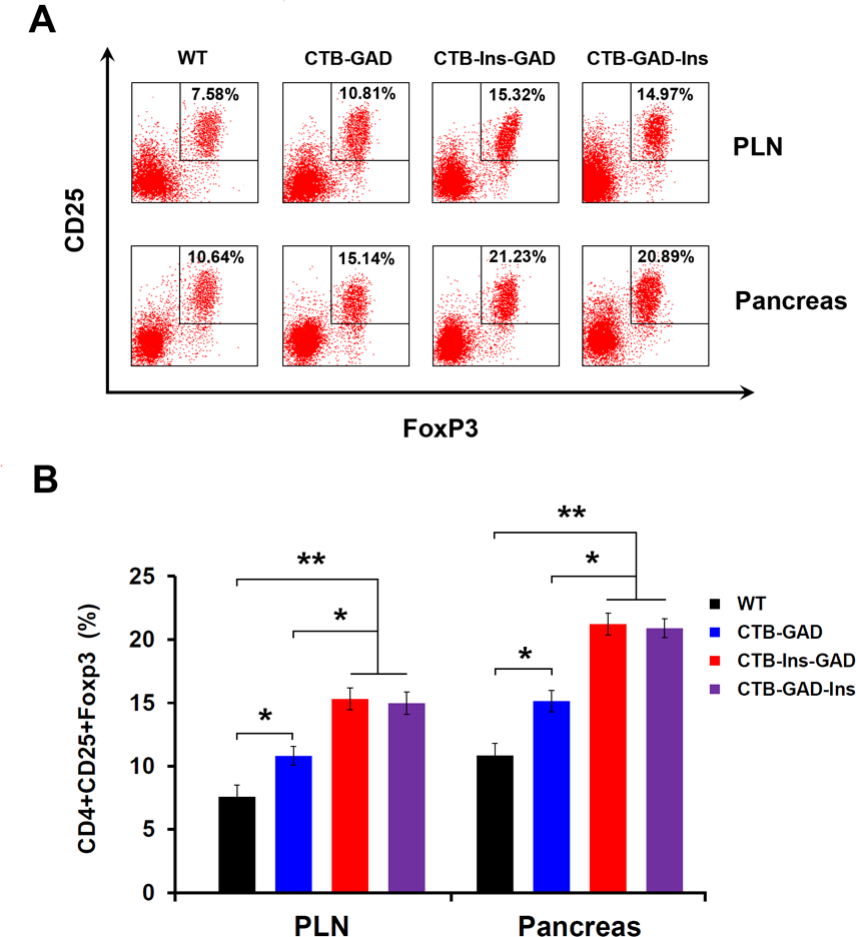
CTB-Ins-GAD and CTB-GAD-Ins induce a greater increase in CD4^+^CD25^+^Foxp3^+^ T cells. (A and B) Lymphocyte (1×10^6^) isolated from CTB-GAD-, CTB-Ins-GAD-, CTB-GAD-Ins-, or WT-fed NOD mice at 10 weeks of age were treated with the mouse regulatory T cell staining kit. These treated lymphocyte were examined using a FACScan flow cytometer to determine CD4^+^CD25^+^Foxp3^+^Treg cell proportions. The gate was set based on CD3^+^CD4^+^. Results are presented as the mean values ± SD. Six mice per group were evaluated in two separate experiments. * p<0.05; ** P<0.01.

We also measured the proportion of CD4^+^CD25^+^Foxp3^+^ T regulatory cells in the peripheral lymph system using Fluorescence Activated Cell Sorter (FACS). Our results showed that mice treated with CTB-Ins-GAD produced a higher proportion of CD4^+^CD25^+^Foxp3^+^ T cells in the pancreas and pancreatic lymph nodes (PLN) CD4^+^ T cell populations compared with the CTB-GAD group (21.23±0.64% vs 15.14±0.71% in the pancreas; 15.32±0.59% vs. 10.81±0.51% in the PLN) (Fig 6A and 6B). A similar result was observed in the CTB-GAD-Ins group. These results indicated that CTB-Ins-GAD specifically increased the proportions of CD4^+^CD25^+^Foxp3^+^ T cells, which may be associated with the suppression of T1D progression in the peripheral lymph system.

**Fig 7:**
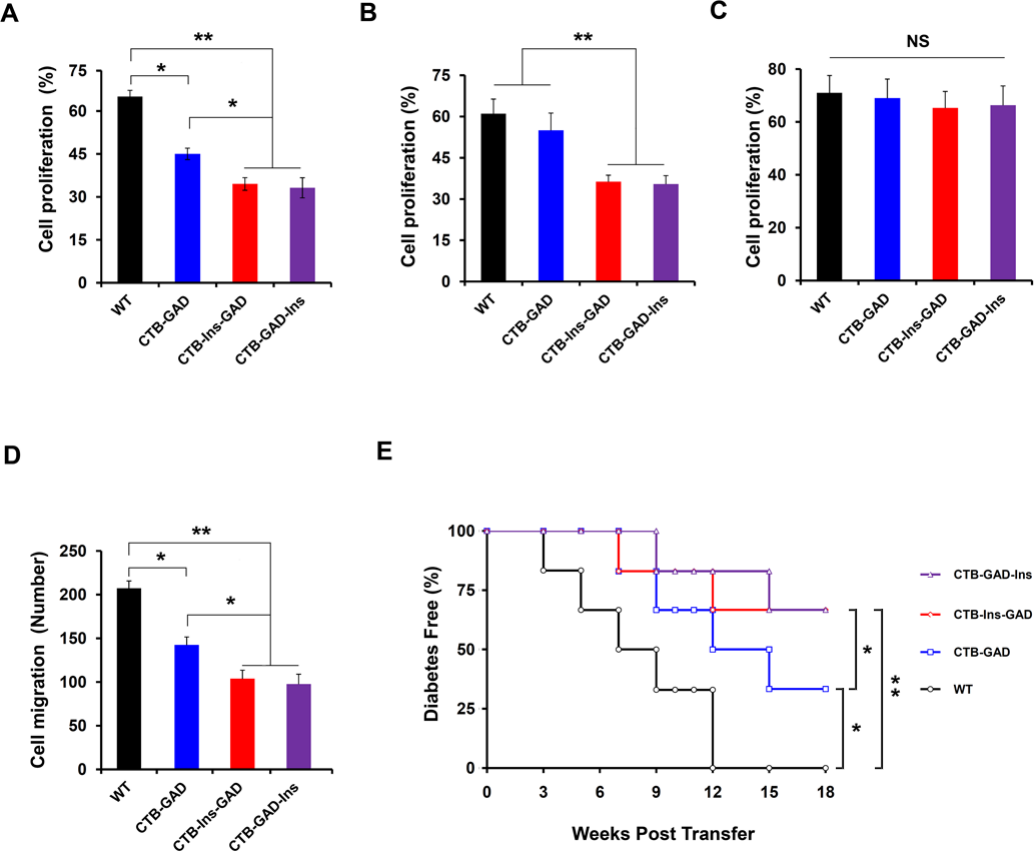
CTB-Ins-GAD and CTB-GAD-Ins treatment induced regulatory T cell differentiation. Proliferation analysis: splenocytes (1×10^7^cells) isolated from CTB-GAD, CTB-Ins-GAD-, CTB-GAD-Ins, WT-fed, and normal mice at 10 weeks of age were cultured in 24-well plates with RPMI 1640 medium and stimulated with GAD65 (A), insulin (B), or PHA (C). Cells were stained immunohistochemically, and the stained cell proportions (representative of proliferative ability) were determined according to the protocol. (D) Migration analysis: splenocytes were cultured in the upper chamber of Transwell plates with 0.6 mL normal culture medium, 150 IU/mL IL-2, 20 μg/ml insulin and 20 μg/ml GAD65 placed in the bottom chamber at 37°C with 5% CO_2_ for 14 h. The migrated cells were fixed, stained, and numbered under the microscope. (E) CTB-Ins-GAD and CTB-GAD-Ins effects in adoptive transfer. Splenocytes (10^6^/well) from CTB-Ins-GAD-, CTB-GAD-Ins-, CTB-GAD-, or WT-immunized mice were mixed with diabetogenic splenocytes (1×10^7^) from diabetic NOD mice, and then co-transferred to 8-week-old NOD/SCID mice (n = 6 for all four groups) using a diabetogenic splenocyte-only injection as a control. The development of diabetes in the recipients was monitored for 18 weeks. Six mice per group were individually tested in two separate experiments. * p<0.05; ** P<0.01; NS: no significant difference.

We also investigated whether CTB-Ins-GAD could specifically suppress GAD65 or insulin-reactive T cells using T-cell proliferation and migration assays. As expected, we observed that mice immunized with CTB-Ins-GAD showed significantly decreased insulin-reactive T cell proliferation compared with mice immunized with CTB-GAD (Fig 7A). Importantly, CTB-Ins-GAD treatment significantly decreased proliferation of splenocytes in response to GAD65 stimulation compared with CTB-GAD treatment (Fig 7B). However, the nonspecific T lymphocyte proliferative response to phytohaemagglutinin (PHA) stimulation was not significantly different among groups (Fig 7C). In the T-cell migration assay, CTB-Ins-GAD treatment significantly decreased the migratory ability of splenocytes compared with CTB-GAD treatment (Fig 7D). A similar result was observed in the CTB-GAD-Ins group. Collectively, these data indicate that CTB-Ins-GAD promotes the suppression of both GAD- and insulin-reactive T cell proliferation and migration.

### Effect of CTB-Ins-GAD and CTB-GAD-Ins in adoptive transfer

To determine whether CTB-Ins-GAD and CTB-GAD-Ins administration induces active immunoregulatory cells to protect against the development of diabetes, we employed an adoptive transfer model. Splenocytes from 10-week-old NOD mice fed CTB-GAD, CTB-Ins-GAD or CTB-GAD-Ins were mixed with diabetogenic splenocytes and intravenously administered to 6- to 8-week-old syngeneic NOD/SCID recipient mice. These mice receiving only diabetogenic splenocytes were used as a positive control. We observed that delaying the onset of diabetes could be adoptively transferred to naive recipients by splenocytes from NOD mice fed CTB-GAD, CTB-Ins-GAD, or CTB-GAD-Ins, albeit with varying effectiveness. As shown in Fig 7E, 100% of NOD/SCID recipient mice receiving splenocytes from WT treated mice developed diabetes. In contrast, only 33.3% of NOD/SCID mice receiving splenocytes from CTB-Ins-GAD or CTB-GAD-Ins developed diabetes, while 66.7% of NOD/SCID mice receiving splenocytes from mice fed CTB-GAD developed diabetes (33.3% vs 66.7%, p<0.05; 33.3% vs 100%, p<0.01; 66.7% vs 100%, p<0.05), respectively, at 18 weeks after transfer These results indicated that CTB-Ins-GAD has a synergistic effect in adoptive transfer by splenocytes from antigen-fed mice, and that oral administration of CTB-Ins-GAD could induce a greater number of regulatory CD4^+^ T cells and suppress diabetogenic T cells.

## Discussion

Although oral administration of these antigens has shown efficacy in preventing T1D in NOD mice [13–15, 21, 23, 24, 35], translation of oral tolerance therapies to clinical applications remains challenging. Successful clinical therapy may be impeded by uncertainty of primary autoantigens, insufficient dosage of antigen, and lack of a suitable mucosal adjuvant or low Treg induction to confer long-term toleration effects [1]. To address these issues, we designed CTB-Ins-GAD with dual antigens expressed in a high-efficiency silkworm bioreactor. Our study indicates that oral administration of CTB-Ins-GAD protects 78% of mice from T1D. Similar oral immunotherapy has been obtained in CTB-GAD-Ins by switching the order of administration of insulin and GAD. This is highly effective for the suppression of T1D with high oral tolerance in NOD mice, compared with a 25–45% suppression of diabetes in most cases [9, 10, 12, 13, 25]. This apparently greater efficacy may be due to the combination of CTB-based multiple antigens with the silkworm bioreactor, thus providing an effective and economic oral immunotherapy against T1D.

First, a combination of multiple antigens is a reasonable and effective immunotherapy strategy against T1D before primary autoantigens, and the mechanisms involved in diabetes have been well elucidated. Although it is clear that multiple islet molecules are the target of autoimmunity in human and animal models, it remains unclear whether any of the target molecules are essential for the destruction of islet beta cells [36, 37]. For example, considerable evidence supports a role for GAD autoreactivity in T1D; however, the role of GAD in diabetogenesis remains unclear [38], and some recent studies doubt the essentiality of GAD as an autoantigen in the pathogenesis of diabetes [39]. Similarly, although overwhelming evidence indicates that insulin is an important target molecule in T1D patients [6], there may be many additional relevant targets [40–42]. We propose that T1D pathology may not be simply downstream of the insulin- or GAD-related autoantigen pathway but could be generated by multiple pathways that interact synergistically. If this is true, it is important to devise a strategy that can simultaneously and effectively target both pathways. This proposal is supported by our results that the oral administration of CTB-Ins-GAD has greater protection against T1D than that of insulin or GAD65 single-antigen treatment. Therefore, although primary autoantigens and their mechanisms in diabetes remain to be explored, a combination of multiple antigens may be a feasible therapeutic approach with complementary or synergistic effects. In addition, our results increase our understanding of the autoantigens involved in the development of T1D.

Combined multiple antigens enhance oral tolerance through combinatorial mechanisms [43]. First, CTB-Ins-GAD may repair the imbalance of Th1/Th2 cells. Previous studies demonstrated that oral administration of insulin or GAD could induce insulin- or GAD-specific Th2-associated antibody subclasses [11, 13, 23, 24, 44]. We show that oral administration of CTB-Ins-GAD may induce a Th2-associated (IgG1) isotype of both GAD65 and insulin-specific antibody subclasses. This result was further supported by the increase in Th2 and decrease in Th1 cells induced by CTB-Ins-GAD. Interestingly, CTB-Ins-GAD induced more GAD65-specific Th2 cells but less GAD65-specific Th1 cells than did CTB-GAD treatment (Fig 5C and 5D). This may be because CTB-Ins-GAD induced a cytokine environment with higher IL-4 and lower IFN-γ levels than those of CTB-GAD treatment (Fig 5E–5H). The differentiation of Th1/Th2 cells dependent on their cytokine environment [45]. The higher IL-4 and lower IFN-γ induced by synergistic of insulin and GAD65 influence naive T cells to eventually become Th2 cells. In contrast, the differentiation of Th1 cells was suppressed. Together, the synergistic of two antigens was more beneficial for the repairing of imbalance of Th1/Th2. Conversely, co-administration of insulin and GAD failed to induce an antigen-specific humoral immune response, nor did it induce any significant transferable suppression, thus accounting for the low efficacy of protection against T1D [25]. Second, CTB-Ins-GAD induced a higher proportion of CD4^+^CD25^+^Foxp3^+^ Treg cells and a stronger inhibition of proliferation and migration in insulin- and GAD-specific reactive T lymphocyte cells. This is also supported by the stronger suppressive effects of Treg cells in adoptive transfer. Together, these results indicated that orally delivered CTB-Ins-GAD induces protection by repairing Th1/Th2 imbalances. The protection may be associated with the increasing CD4^+^CD25^+^Foxp3^+^ Treg cells.

Second, our study indicates that the CTB is essential for the high protective effect, even when using multiple antigens. Multiple studies indicate that CTB, IL-4, or IL-10 enhance the therapeutic efficacy against T1D [14, 46–48]. Strikingly, oral administration of insulin or GAD has little therapeutic effect on T1D without CTB, IL-4, or IL-10 [11, 18]. Previous studies demonstrated that the combination of 1 mg human insulin and 0.5 mg GAD was more protective than oral GAD alone; however, its suppressive efficacy was only 25% [25]. Importantly, it did not induce an antigen-specific humoral immune response, possibly because it lacks the mucosal immune response system-targeting capability of the CTB carrier molecule. Another study showed that immunization of NOD animals with syngeneic islet lysates results in a significant delay in diabetes onset (P < 0.001) compared with non-immunized controls [49]. We believe that islet lysates contain some cytokines, such as IL-10 and IL-4, or multiple antigens are correlated with the protective effect. It is possible that the efficacy of CTB-Ins-GAD is improved if combined with other cytokines such as IL-4. Together, mucosal adjuvants are crucial for successful oral immunotherapy on T1D using multiple antigens.

Lastly, the potential success of oral immunotherapy depends not only on the identification of important autoantigens and the usage of mucosal adjuvants, but also on the capacity to produce sufficient and affordable autoantigens for clinical applications. Edible plants are an attractive approach to produce antigens [21, 28, 29, 50]; however, the expression levels of transgenic proteins may be too low for an oral tolerizing effect, ranging from 0.002–0.4% of total soluble proteins [21, 28]. The use of a silkworm bioreactor for oral antigen immunotherapy has considerable clinical appeal due to its high expression capacity, simplicity of production, and delivery, low cost, and safety to humans. Our group has produced recombinant CTB-Ins and CTB-InB at levels reaching 0.4–0.97 g/l hemolymph in a silkworm expression system [12, 13, 33]. Using the silkworm bioreactor, we have developed the first rhGM-CSF oral drug, which is undergoing clinical trials [34]. In the present study, the CTB-Ins-GAD expression levels reached 0.49 g/l hemolymph in silkworm. Thus, the silkworm bioreactor is a highly efficient production and delivery system for oral antigens for clinical applications in the near future.

In summary, our study demonstrated that oral administration of CTB-Ins-GAD more effectively suppresses T1D than does insulin or GAD65 single-antigen treatment. The resulting synergistic beneficial effect was achieved through repaired Th1/Th2 imbalance, and which may be associated with the increased numbers of CD4^+^CD25^+^Foxp3^+^ Treg cells. In combination with the high-efficiency silkworm bioreactor, the oral tolerance induced by multiple antigens provides an effective and economic approach for the prevention of human T1D and other autoimmune diseases.

## Supporting Information

S1 Table Seven primers synthesized for the construction of the fusion genes. (DOC)
