# Supplementary material for: Oral Administration of Silkworm-Produced GAD65 and Insulin Bi-Autoantigens against Type 1 Diabetes
Source: PLoS One. 2016 Jan 19;11(1):e0147260. doi: 10.1371/journal.pone.0147260 (PMC4718521; doi:10.1371/journal.pone.0147260)
Supplement: S1 Table — (DOC) [file pone.0147260.s001.doc]

**S1 Table. Seven primers synthesized for the construction of the fusion genes.**

| NO. | Sequence | Length |
| --- | --- | --- |
| P1 | 5′-CG*GGATCC*ATGATTAAATTAAAATTTGG-3′ | 28bp |
| P2 | 5′-GGGGCCGGGGCCGTTGCAGTAGTTCTCCAGCTGGTAGAGG-3′ | 40bp |
| P3 | 5′-GGCCCCGGCCCCCCGGTTATCAAAGCTCG-3′ | 29bp |
| P4 | 5′-GG*GAATTC*TTAAACCATGGTAGTACCGTA-3′ | 29bp |
| P5 | 5′-GGGGCCGGGGCCAACCATGGTAGTACCG-3′ | 28bp |
| P6 | 5′-*GGCCCCGGCCCC*TTTGTGAACCAACACCT-3′ | 29bp |
| P7 | 5′-GG*GAATTC*TTAGTTGCAGTAGTTCTCCAGCTGGTAGAGG-3′ | 39bp |
